# Leakage Characteristics of Dual-Cannula Fenestrated Tracheostomy Tubes during Positive Pressure Ventilation: A Bench Study

**DOI:** 10.1155/2016/9272865

**Published:** 2016-03-17

**Authors:** Thomas Berlet, Mathias Marchon

**Affiliations:** ^1^Department of Intensive Care Medicine, Inselspital-Bern University Hospital, 3010 Bern, Switzerland; ^2^Department of Anaesthesiology and Pain Therapy, Inselspital-Bern University Hospital, 3010 Bern, Switzerland

## Abstract

This study compared the leakage characteristics of different types of dual-cannula fenestrated tracheostomy tubes during positive pressure ventilation. Fenestrated Portex® Blue Line Ultra®, TRACOE® twist, or Rüsch® Traceofix® tracheostomy tubes equipped with nonfenestrated inner cannulas were tested in a tracheostomy-lung simulator. Transfenestration pressures and transfenestration leakage rates were measured during positive pressure ventilation. The impact of different ventilation modes, airway pressures, temperatures, and simulated static lung compliance settings on leakage characteristics was assessed. We observed substantial differences in transfenestration pressures and transfenestration leakage rates. The leakage rates of the best performing tubes were <3.5% of the delivered minute volume. At body temperature, the leakage rates of these tracheostomy tubes were <1%. The tracheal tube design was the main factor that determined the leakage characteristics. Careful tracheostomy tube selection permits the use of fenestrated tracheostomy tubes in patients receiving positive pressure ventilation immediately after stoma formation and minimises the risk of complications caused by transfenestration gas leakage, for example, subcutaneous emphysema.

## 1. Introduction

Percutaneous dilatational tracheostomy (PDT) is frequently performed in the intensive care unit [1]. Common indications for PDT are a prolonged duration of and gradual weaning from mechanical ventilation, protection of the tracheobronchial tree in patients at risk for aspiration, and the requirement to frequently access the respiratory tract for diagnostic or therapeutic purposes [2].

As soon as the need for high-level respiratory support subsides, trials of spontaneous breathing are usually introduced. During the rehabilitation phase the initial configuration of a tightly sealed tracheostomy tube during positive pressure ventilation is changed to cuff deflation during episodes of spontaneous breathing [3]. Here, the use of a fenestrated tracheostomy tube in combination with a fenestrated inner cannula is advantageous, because the work of breathing is minimised by the low airflow resistance afforded by these tubes [4, 5]. Even more importantly, swallowing, communication, postural stabilisation, and weight-bearing ability are better supported by dual-cannula fenestrated tracheostomy tubes, particularly when a speaking valve is mounted [6–9].

Following the publication of reports of subcutaneous emphysema and pneumothorax developing in connection with the use of dual-cannula fenestrated tracheostomy tubes for PDT, many authors advised against the use of these types of tubes in patients who require positive pressure ventilation [1, 10–12].

We hypothesised that the risk of surgical emphysema formation is related to the degree of air leakage through the fenestrations. We therefore performed a bench study to quantify and compare the leakage characteristics of different types of commonly used dual-cannula fenestrated tracheostomy tubes under varying conditions. A pseudotrachea was used to study ex vivo the performance of tracheostomy tubes. Our aim was to derive recommendations regarding the safe use of fenestrated tracheostomy tubes immediately after tracheostomy formation.

## 2. Materials and Methods

**Figure 1:**
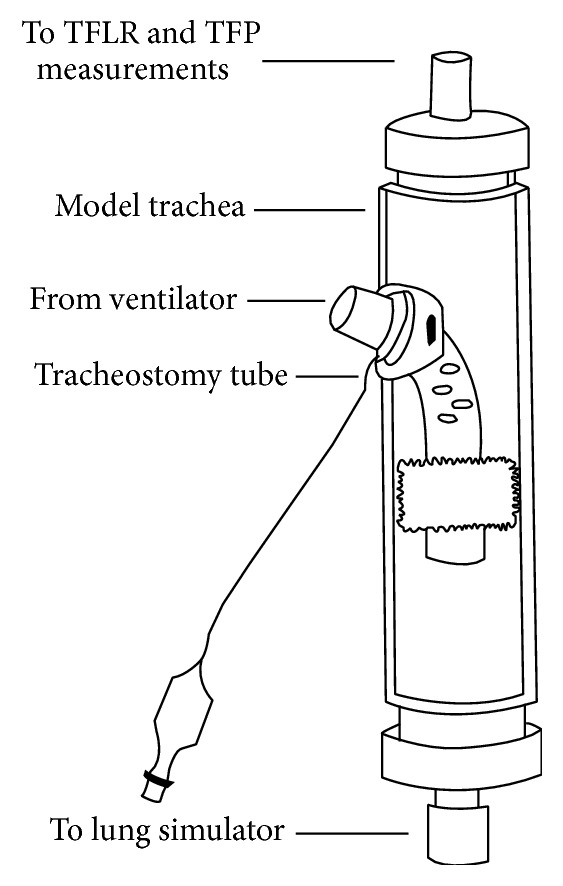
Schematic drawing of tracheostomy simulator with a fenestrated tracheostomy tube sited. TFLR: transfenestration leakage rate; TFP: transfenestration pressure.

We built a tracheostomy simulator comprising a polyethylene tube that represented the trachea (length, 20 cm; internal diameter, 25 mm). A 10 mm side hole was fashioned at one-third the length of the tubing; it represented the tracheostomy (Figure 1). Different types of fenestrated tracheostomy tubes with nonfenestrated inner cannulas were studied (Table 1).

A tracheostomy tube to be tested was inserted through the tracheostomy, and the cuff was inflated. A seal was fashioned around the tracheostomy insertion site using rubber washers and putty. Leak testing was performed prior to each experiment to ascertain the leak tightness of both the tracheostomy tube cuff within the trachea and the insertion site of the tracheostomy tube. The trachea simulator was then placed upright into a laboratory stand. The lower (“bronchial”) portion of the artificial trachea was connected to a single compartment lung simulator (LS-122; Medishield, Harlow, Essex, UK), and the upper (“pharyngeal”) portion of the artificial trachea was connected to a 2.3-l anaesthesia breathing bag so that any gas escaping through the fenestrations of the tracheostomy tube could be collected. Polyvinylchloride fittings and nonexpandable polyethylene tubing were used for all connections. A SERVO-i® ventilator (Maquet, Rastatt, Germany) was connected to the tracheostomy tube. The ventilator and lung simulator compliance settings were varied as described in Table 2.

Experiments were run at either 21°  ± 1°C or 37°  ± 1°C. For the latter experimental runs, a polyethylene hood was fashioned to encase the laboratory stand holding the tracheostomy simulator. Preheated air, generated by a Bair Hugger® temperature management device (3M, Rüschlikon, Switzerland), was fed into the hood. The temperature of the tracheostomy simulator was continuously monitored with a digital thermometer (TFA®; Conrad Electronic SE, Hirschau, Germany).

Two types of measurements were obtained for each combination of ventilator and lung simulator settings and temperatures. The first measurement was the transfenestration pressure (TFP): the pharyngeal end of the trachea was occluded by clamping the connecting tubing, and the build-up of pressure in the space above the tracheostomy tube was measured with a digital manometer (PCE-P01; PCE Instruments, Meschede, Germany) until a steady state was reached. The second measurement was the transfenestration leak rate (TFLR) (minute ventilation fraction). Prior to each experimental run, the anaesthesia bag was thoroughly emptied and gas escaping through the fenestrations of the tracheostomy tube was collected into the anaesthesia breathing bag for 1–3 min, depending on the magnitude of the leakage. The volume of the collected gas was measured using the water displacement method. To distinguish between the amount of gas leaking through the fenestration and the amount of gas leaking through the connection site of the inner and outer cannula, the difference between the inspiratory and expiratory minute volumes measured by the ventilator and the TFLR was calculated. All experiments were run in duplicate. Measurements were repeated if corresponding pressure measurements differed by >5% or volume measurements differed by >5% and >10 mL·min^−1^.

Statistical analysis was performed using the StatPlus® 8 software package (AnalystSoft Inc., Alexandria, VA, USA). Data are presented as individual measurements. The Mann-Whitney test was used for comparisons of nonnormally distributed continuous data, and the Wilcoxon matched-pairs test was used for comparisons of nonnormally distributed paired data. Spearman's rank order correlation coefficient was used to assess the relationships between continuous variables. All tests of statistical significance were two-sided. *P* values of <0.05 were considered statistically significant.

## 3. Results

**Figure 2:**
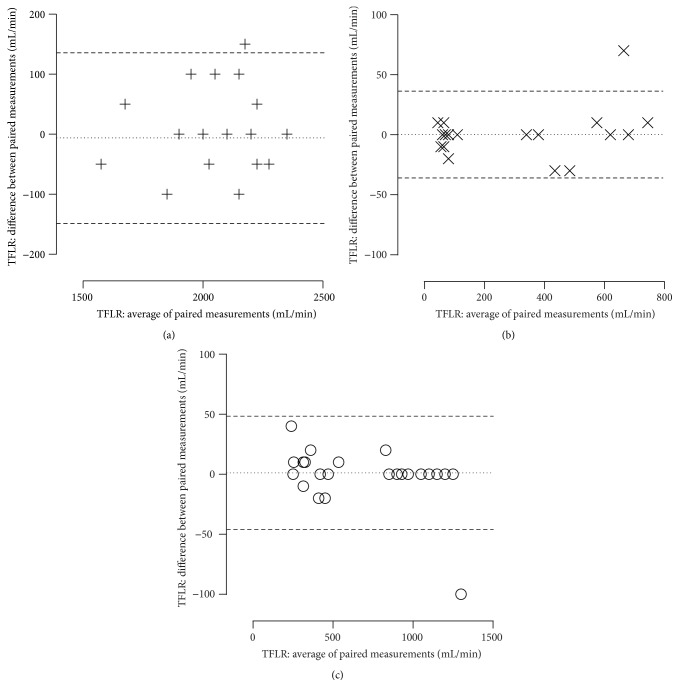
Bland-Altman plot of paired transfenestration leakage rates of Portex Blue Line Ultra (+), TRACOE twist (×), or Rüsch Traceofix (○) tracheostomy tubes. TFLR: transfenestration leakage rate.

The reliability of the experimental setup as assessed by the Bland-Altman method is depicted in Figures 2(a)–2(c). One hundred and thirty-six of 144 paired TFLR measurements differed by no more than 5% or 10 mL·min^−1^.

Differences in TFLR and TFP were observed between different types of tracheostomy tubes exposed to identical experimental conditions. Additionally, variations in TFLR and TFP were observed for each tracheostomy tube as the experimental conditions were modified.

TFLR ranged from 0.2% to 67.6% of minute ventilation, and TFP ranged from 1 to 16 mbar across the entire range of investigated combinations of types of tracheostomy tubes, temperatures, ventilation modes, and compliance settings.

**Figure 3:**
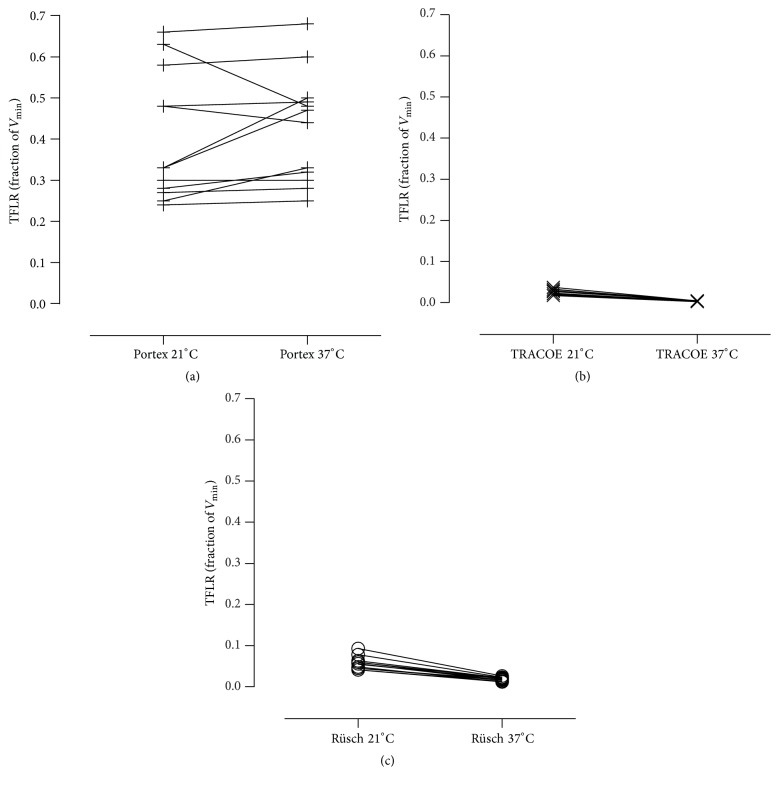
Transfenestration leakage rates of Portex Blue Line Ultra (+), TRACOE twist (×), or Rüsch Traceofix (○) tracheostomy tubes. Measurements were performed at 21 ± 1°C or 37 ± 1°C. TFLR: transfenestration leakage rate; *V*_min_: minute ventilation.

Figures 3(a)–3(c) illustrate the variations in TFLR at different temperature settings. Raising the temperature of the experimental setup from 21°C ± 1°C to 37°C ± 1°C resulted in no statistically significant changes in TFLR when using the Portex Blue Line Ultra tracheostomy tubes (*P* = 0.403). In contrast, TFLR decreased significantly when using the TRACOE twist and Rüsch Traceofix tubes (*P* = 0.0022).

Because tracheostomy tube's performance at body temperature is relevant to clinical practice, the following results refer to experiments that were performed at 37 ± 1°C.

**Figure 4:**
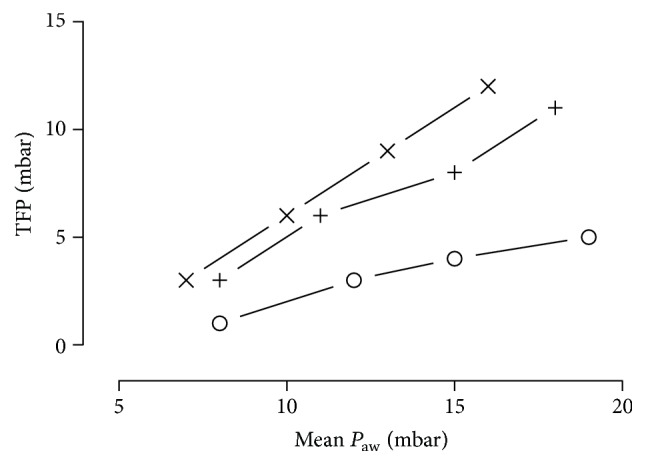
Transfenestration pressures of Portex Blue Line Ultra (+), TRACOE twist (×), or Rüsch Traceofix (○) tracheostomy tubes plotted against increase in mean airway pressures. Measurements were obtained in volume-controlled ventilation (ventilator rate: 15 breaths·min^−1^, tidal volume: 450 mL) at 37 ± 1°C. Lung simulator compliance: 20 mL·mbar^−1^. TFP: transfenestration pressure; *P*_aw_: mean airway pressure.

Figure 4 illustrates the relationship between the mean airway pressure and TFP. The correlation coefficient was 0.98 for the Rüsch Traceofix tubes, 0.99 for the TRACOE twist tubes, and 1.0 for the Portex Blue Line Ultra tubes.

**Figure 5:**
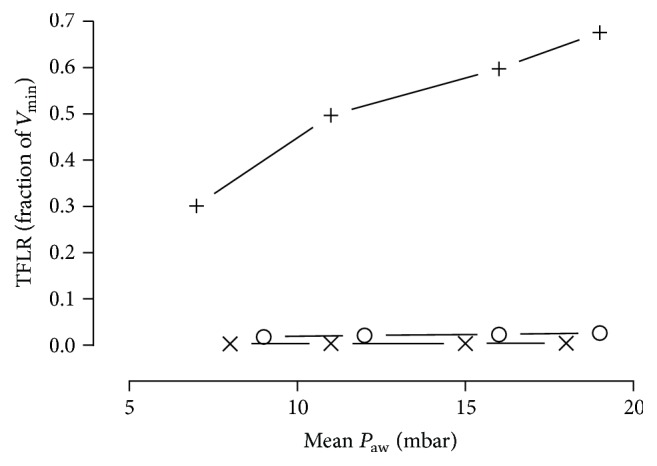
Transfenestration leakage rates of Portex Blue Line Ultra (+), TRACOE twist (×), or Rüsch Traceofix (○) tracheostomy tubes plotted against increase in mean airway pressures. Measurements were obtained in volume-controlled ventilation (ventilator rate: 15 breaths·min^−1^, tidal volume: 450 mL) at 37 ± 1°C. Lung simulator compliance: 20 mL·mbar^−1^. TFLR: transfenestration leakage rate; *P*_aw_: mean airway pressure; *V*_min_: minute ventilation.

Figure 5 illustrates the relationship between the mean airway pressure and TFLR. The correlation coefficient was 0.89 for the TRACOE twist tubes, 0.98 for the Portex Blue Line Ultra tubes, and 0.99 for the Rüsch Traceofix tubes.

**Figure 6:**
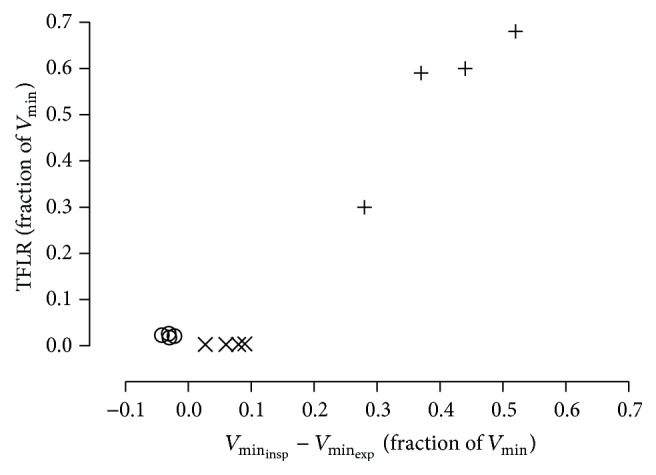
Loss of delivered minute volume of Portex Blue Line Ultra (+), TRACOE twist (×), or Rüsch Traceofix (○) tracheostomy tubes plotted against transfenestration leakage rate measured at PEEP settings of 3, 6, 9, and 12 mbar. Measurements were obtained in volume-controlled ventilation (ventilator rate: 15 breaths·min^−1^, tidal volume: 450 mL) at 37 ± 1°C. Lung simulator compliance: 20 mL·mbar^−1^. TFLR: transfenestration leakage rate; *V*_min_insp__: inspiratory minute ventilation; *V*_min_exp__: expiratory minute ventilation.

By comparing the ratio of TFLR to the difference in the inspiratory and expiratory minute volume as measured by the ventilator, an additional source of leakage at the interface of the inner and outer cannulas was identified in the TRACOE twist tube (Figure 6).

Switching from the volume-controlled ventilation mode to the pressure-controlled ventilation mode resulted in no statistically significant variation in TFP or TFLR for any make of tracheostomy tube.

Changes in static compliance did not significantly alter TFP or TFLR for any make of tracheostomy tube.

## 4. Discussion

In this study, the sources and degree of leakage of different types of tracheostomy tubes during positive pressure ventilation, the impact of the tube design, and the variability of tracheostomy tube's performance at different temperatures were investigated for the first time.

We found substantial variations in TFLR and TFP for different types of fenestrated tracheostomy tubes when used in combination with nonfenestrated inner cannulas. The leakage rates of the best performing tubes did not exceed 3.5% of the delivered minute volume. When tested at body temperature, the leakage rates of these tracheostomy tubes dropped even further to <1%. The tracheal tube design was the main factor that determined the leakage characteristics. Two features of the cannula design were found to be associated with the lowest TFLR and TFP: connection of the ventilator catheter mount to the inner cannula and a tightly sealed interface of the inner and outer cannulas. The Blue Line Ultra tracheostomy tube is designed to connect to the ventilator catheter mount via the outer cannula; it yielded the highest TFLR and TFP. The TRACOE twist tube had low TFLR but a significant degree of leakage at the interface of the inner and outer cannulas. The Rüsch Traceofix tracheostomy tube had low TFLR and TFP and a tight seal at the cannula interface. The ventilation mode and lung compliance had little impact on the leakage characteristics.

The cause of subcutaneous emphysema after PDT and insertion of dual-cannula fenestrated tracheostomy tubes is the tracking of air between the nonfenestrated inner cannula and the fenestrated outer cannula with subsequent leakage through the fenestrations [10, 12]. If the air cannot escape through the glottis or the tracheostomy wound, it tracks into the soft tissue of the neck, particularly if the fenestrations abut the subcutaneous tissue.

Mostert and Stuart [10] gave the first report of a patient who developed subcutaneous emphysema while being mechanically ventilated through a newly inserted fenestrated Portex Blue Line Ultra tracheostomy tube. Following the publication of that case report, the manufacturer issued a warning to confirm the correct position of the fenestration after the procedure. A review of the tracheostomy tube's design implicated in the critical incident was envisaged; however, the design of the Portex Blue Line Ultra tracheostomy tube with regard to the position of the fenestrations appears to have since remained unchanged.

Fikkers et al. [12] presented a review of the literature on emphysema and pneumothorax after percutaneous tracheostomy. Sixty-six cases of pneumothorax or subcutaneous emphysema were identified; two of these (3%) were caused by extraluminal misplacement of the fenestrations of the tracheotomy tubes. These authors performed a bench study using a fenestrated Shiley tracheostomy tube fitted with a nonfenestrated inner tube. The leakage rate, as detected by the difference between the inspiratory and expiratory tidal volumes during pressure-controlled ventilation, reached 2780 mL·min^−1^. The authors also measured the size of the gap between the inner circumference of the outer cannula and the outer circumference of the inner cannula at 0.14 mm. In the present study, comparable leakage rates were found in the Blue Line Ultra tracheostomy tube.

Orme and Welham [11] reported a case of subcutaneous emphysema that occurred with the use of a fenestrated Portex Blue Line Ultra tracheostomy tube with a nonfenestrated inner cannula. Two faults contributed to the emphysema formation: malpositioning of the fenestrations of the tracheostomy tube and undetected partial detachment of the inner cannula. These problems caused the surrounding tissues to be fully exposed to the pressure generated by the ventilator. The authors called for discontinuation of the use of fenestrated cannulas early after tracheostomy. Their report corroborates our findings of the significance of the tightness of the seal at the interface between the inner and outer cannulas.

In 2008, the UK Intensive Care Society [13] recommended against the use of fenestrated tracheostomy tubes early after stoma formation. This advice was carried forward to the current version of the guidelines [1]. Powell et al. [14] conducted a survey of the use of nonfenestrated versus fenestrated tracheostomy tubes in UK intensive care units. Eight of eight units that used fenestrated tracheostomy tubes reported the occurrence of surgical emphysema in any of their patients. The authors interpreted this as a powerful indicator of the frequent causation of subcutaneous emphysema by the use of fenestrated tubes.

Weaning from mechanical ventilation and from the tracheostomy itself is a challenging, often drawn out task, particularly after long-term ventilator support. The ability to communicate verbally is an important step towards reestablishing patient autonomy and quality of life [7, 15, 16]. Cuff deflation of the tracheostomy tube is performed not only to facilitate spontaneous breathing and promote swallowing but also, and arguably more importantly, to enable communication.

During spontaneous breathing through a tracheostomy tube, airflow resistance should be decreased to minimise the work of breathing [4]. In this scenario, the use of a fenestrated tracheostomy tube is preferable to the use of a nonfenestrated tube [5]. Communication is aided by the use of a fenestrated tube [2]. If a speaking valve is attached, its tolerance can be increased by the additional airflow through the fenestrations of the tracheostomy tube [3]. Engaging vocal cord function improves postural stability and weight-bearing ability. This is best achieved with a cuff-deflated fenestrated tracheostomy tube used in combination with a speaking valve [9]. However, care must be taken not to negate the benefits of reduced airflow resistance of fenestrated tracheostomy tubes by malpositioning of the fenestrations [17].

Abandoning the use of fenestrated tracheostomy tubes reduces both the speed and the efficiency of the rehabilitation process, because the advantages of these types of tubes will be unavailable until a change of tracheostomy tube. This can only be safely performed several days after the initial procedure; it requires resources and carries certain risks [17].

Our work provides insight into the benefits of using particular types of fenestrated tracheostomy tubes and can be used in the selection of a suitable tracheostomy tube. In devising a pseudotrachea we built on the experience reported by Hussey and Bishop of the use of a model trachea to study tracheostomy tube's performance [5]. We believe that our experimental setup offered sufficient fidelity to serve as a surrogate for an in vivo tracheostomy scenario.

Our study could be criticised for not including the entire range of commercially available tracheostomy tubes. Because we aimed to explore not only the implication of the tube design but also the impact of temperature, ventilator settings, and lung compliance, we made a conscious decision to limit the scope of the study to a selection of widely used tracheostomy tubes.

The thermal behaviour of plastic materials provides a possible explanation for our observation of a significant improvement in leakage rates when tracheostomy tubes were tested at body temperature. Tracheostomy tubes are made from polyvinylchloride or polyurethane, and inner tubes are made from polypropylene. These materials are thermoplastics: they expand and become more pliable as temperature rises [18]. Temperature-dependent expansion of both the outer cannula and inner cannula will narrow the gap between these two components, thus reducing the potential of air tracking toward the fenestration. The increase in pliability may have a synergistic effect: the pressure exerted by the inflated cuff may push the outer cannula further toward the inner cannula, thus further narrowing the gap between the two. As a result of the overall reduction in space available for air leakage, the gas flow will diminish, significantly reducing TFP and particularly TFLR.

Little is known about the impact of the thermoplastic characteristics of the various plastic materials used on tracheostomy tube performance [19]. Further research should be performed to explore this aspect in more detail with the aim of further optimisation of the design of fenestrated tracheostomy tubes.

## 5. Conclusion

Transfenestration gas leakage of fenestrated tracheostomy tubes is highly variable when these tubes are used in combination with nonfenestrated inner cannulas and exposed to positive pressure ventilation. In vitro leakage testing enables the identification of fenestrated tracheostomy tubes that are suitable for immediate use after stoma formation in patients expected to benefit from early trials of spontaneous breathing and rehabilitation of swallowing, communication, and mobilisation.

## Figures and Tables

**Table 1:** Types and specifications of tracheostomy tubes tested.

Type	Manufacturer	Size	Inner cannula Internal diameter	Outer cannula Outer diameter
Portex Blue Line Ultra	Smith Medical, Grasbrunn, Germany	8	6.5 mm	11.9 mm
TRACOE twist	Tracoe Medical, Nieder-Olm, Germany	8	8.0 mm	11.4 mm
Rüsch Traceofix	Teleflex, Kernen, Germany	8.5	7.0 mm	10.3 mm

**Table 2:** Ventilator and lung simulator compliance settings.

Variable	Settings
Ventilation mode	Volume-controlled Pressure-controlled
Ventilator rate (breaths·min^−1^)	15
Tidal volume (mL)	450 ± 5%
Inspiration : expiration ratio	1 : 2
Positive end-expiratory pressure (mbar)	3, 6, 9, 12
Lung simulator compliance (mL·mbar^−1^)	20, 50
Lung simulator resistance (mbar·mL^−1^·sec^−1^)	5
